# Reduced-temperature pasteurization of apple juice using a flow-through sonicator

**DOI:** 10.1016/j.ultsonch.2026.107926

**Published:** 2026-06-21

**Authors:** Sara Maghami, Kimmo Rumpunen, Örjan Johansson

**Affiliations:** a Luleå University of Technology, Department of Civil, Environmental and Natural Resources Engineering, Sweden; b Swedish University of Agricultural Sciences, Department of Plant Breeding, Sweden

**Keywords:** Acoustic cavitation, Hydrodynamic cavitation, Reduced temperature pasteurization, Thermosonication, Dual-frequency excitation

## Abstract

Thermal pasteurization is widely applied to ensure the microbial safety of fruit juices; however, it can degrade heat-sensitive nutritional and sensory attributes. In this study, a reduced-temperature processing approach based on combined acoustic and hydrodynamic cavitation was investigated using a flow-through sonication system. The sonicator design was validated through both simulation and experimental results. It enables an efficient combination of acoustic and hydrodynamic cavitation, improving energy transfer, pressure localization, generation of transient cavitation, and increased field complexity. The effects of excitation mode (single and dual-frequency), temperature (30–55 °C), and treatment time were evaluated in terms of microbial inactivation, microstructural modification, and physical stability. Dual-frequency excitation at 50–55 °C (DF-4) showed the highest performance, achieving a log reduction of 3.6 for yeast, 2.7 for mold, and 2.8 for aerobic microorganisms as a hygiene indicator after 450  s efficient time. SEM observations confirmed progressive cellular disruption, while sedimentation tracking indicated improved stability due to reduced particle size and improved dispersion. The proposed approach improves microbial inactivation and modifies the microstructure and stability of the juice, highlighting its potential for processing at reduced-temperatures of apple juice.

## 1 Introduction

Fruit and vegetable juices are widely valued for their vitamins, minerals, and antioxidant-rich bioactive compounds[1], [2], [3]. However, due to their high water activity and nutrient content, they are highly susceptible to microbial contamination, making safety assurance and shelf-life extension critical challenges. Thermal pasteurization remains the dominant industrial method, typically implemented through low-temperature long-time (LTLT) or high-temperature short-time (HTST) processes. According to the U.S. Food and Drug Administration (FDA), juice pasteurization must achieve at least a 5-log reduction of the target pathogenic microorganism[4], Arya et al. [5]. Among fruit juices associated with foodborne illnesses, apple juice is the most reported [6].

Various combinations of time and temperature have been investigated for juice pasteurization[7], [8], [9], [10], [11]. Pasteurization time can vary from a few seconds to up to one hour, depending on operating conditions such as pressure, temperature (typically above 60 °C), and the methodology used. Previous studies [12] have demonstrated effective enzyme inactivation, particularly of polyphenol oxidase (PPO), resulting in improved cloud and color stability. Despite these advantages, thermal processing can negatively affect nutritional and sensory quality due to degradation of heat-sensitive compounds [13], [14], [15], [16].

To overcome these limitations, several non-thermal and hybrid technologies have been explored. Among them, cavitation-based processes have attracted considerable attention due to their ability to intensify physicochemical effects in liquid systemsMilly et al. [17], [18], [19]. Cavitation can be induced through acoustic or hydrodynamic means, producing localized high-temperature and high-pressure conditions, along with intense shear forces and microturbulence. These effects contribute to enhanced microbial inactivation, enzyme degradation, and mass transfer [20], [21], [22]. Compared with other emerging technologies such as high-pressure processing (HPP) [23], [24]and pulsed electric field processing (PEF)[25], [26], cavitation-based systems offer advantages including enhanced controllability and compatibility with continuous flow operationArya et al. [5], [27], [28].

High-intensity ultrasound, as a key method for inducing acoustic cavitation, has been widely investigated in juice processing [29], [30], [31], [32], [33]. It promotes structural and cellular modifications within the medium while maintaining relatively low bulk temperatures [34], [35].

However, when applied at sublethal temperatures, ultrasound alone typically results in insufficient microbial reduction, often failing to meet the 5-log requirement. Therefore, it is commonly combined with mild heat (thermosonication) to improve the inactivation efficiency. For example, treatment at 60 °C combined with ultrasound has been reported to achieve approximately a 3-log reduction in *Salmonella Senftenberg*, while thermal treatment alone at the same temperature results in only approximately a 0.5-log reduction, highlighting the synergistic effect of ultrasound and heat [6].

Hydrodynamic cavitation remains a challenge by sonicator design, despite the advantages of flow-based configurations such as Venturi devices, which support continuous processing and scaling Arya et al. [36],Gevari et al. [37]. Variations in flow systems can enhance cavitation generation; however, limitations persist in terms of energy transfer efficiency and precise control of cavitation intensity, particularly in complex juice matrices. Additionally, most of the reported studies are based on laboratory-scale systems operating at high power densities, limiting their direct industrial applicability [38], [39], [40].

Recent numerical and theoretical studies have shown that multi-frequency ultrasound can alter bubble dynamics more effectively than conventional single-frequency excitation. In particular, dual-frequency excitation has been reported to reduce the inertial cavitation threshold compared with single-frequency excitation, while triple-frequency excitation may further decrease the threshold under optimized frequency combinations. This enhancement is attributed to the superposition of acoustic fields, increased pressure waveform complexity, and nonlinear bubble responses involving combination and subharmonic resonances [41], [42]. However, the cavitation response is strongly dependent on the selected frequency combination, pressure amplitude, bubble size, and medium properties, indicating that multi-frequency excitation does not universally enhance cavitation but requires appropriate acoustic design [41], [43]. In dual-frequency fields, additional coupling parameters such as frequency difference, phase difference, and power distribution can further influence the radial oscillation and collapse behavior of cavitation bubbles [42], [44]. These mechanisms provide a theoretical basis for using dual-frequency stimulation to intensify cavitation activity in flow-through liquid processing systems.

The present study addresses these challenges by integrating structural–acoustic modeling into the design of a low-temperature cavitation-based processing system. A flow-through sonication configuration is developed that incorporates hydrodynamic cavitation generated through a Venturi nozzle in combination with an acoustically excited and resonance-amplified flow-through zone [45]. In particular, a dual-frequency excitation strategy is employed to enhance cavitation activity through the interaction of multiple acoustic fields and their harmonics. The system is designed to intensify bubble-collapse dynamics and improve energy transfer efficiency under reduced-temperature conditions. The main objective of the study is to investigate the combined effects of acoustic and hydrodynamic cavitation on microbial inactivation and microstructural modification of fruit juice, with particular emphasis on process intensification (signal characteristics, temperature, treatment time) and scalability.

## 2 Effects of cavitation on microstructure and microbial inactivation

Fruit and vegetable juices can be described as complex multiphase polydisperse systems, comprising a dispersed particulate phase—consisting of cells, cell fragments, and insoluble biopolymers—suspended within a continuous liquid phase containing dissolved sugars, organic acids, and soluble polysaccharides [46], [47]. The application of ultrasound alters the structural characteristics of this system by promoting particle disruption, improved dispersion, and enhanced phase interactions. Under sufficiently intense conditions, these effects contribute to microbial cell disruption and inactivation [48].

The collapse of cavitation bubbles generates localized pressure shocks and shear stresses that disrupt cellular structures and promote the release of intracellular components into the surrounding medium[49], [50].

The intensified bubble activity expected under dual-frequency excitation can further promote localized shear forces, microstreaming, and collapse-induced pressure fluctuations [41], [42], thereby contributing to microbial cell damage and microstructural modification.

With increasing ultrasonic energy, progressive fragmentation and dispersion of cellular material occur, leading to significant modification of the juice microstructure and a shift in particle size distribution toward smaller sizes with increased surface area. This results in enhanced particle–particle and particle–serum interactions and a more homogeneous dispersion. Ultrasound reduces particle size by disrupting aggregates and cellular structures, while the release of intracellular components modifies the composition of the continuous phase. These combined effects influence both interparticle interactions and rheological properties. Depending on the intensity of the processing, the release of soluble polysaccharides can increase viscosity, whereas extensive structural degradation can lead to a reduction in viscosity [51], [52], [53].

These structural changes directly influence sedimentation behavior and physical stability. According to Stokes’ law [35], the particle settling velocity can be expressed as

$$v=\frac{2}{9}\frac{r^{2}\left(\rho_{p}-\rho_{f}\right)g}{\mu }$$

where v is the settling velocity, r is the particle radius, $\rho_{p}$ and $\rho_{f}$ are the densities of the particle and fluid, respectively, g is the gravitational acceleration, and μ is the dynamic viscosity of the fluid. Although Stokes’ law strictly applies to dilute systems of spherical particles under laminar flow conditions, it provides a useful framework for qualitatively describing sedimentation behavior in complex juice systems.

Consequently, reduction in particle size, together with changes in viscosity, leads to a significant decrease in the settling velocity, allowing particles to remain suspended for longer periods and improving suspension stability compared to untreated samples. However, excessive processing may induce secondary aggregation, increasing the effective particle size and potentially improving sedimentation and affecting turbidity. Overall, these modifications contribute to improved textural attributes, including enhanced consistency and mouthfeel [54], [55], [56].

From a microbiological perspective, ultrasound induces cell damage through both mechanical effects, such as disruption of the membrane and shear stress, and chemical effects associated with radical formation. Ultrasound has been shown to reduce the growth of spoilage microorganisms, including yeast and molds, as well as pathogenic bacteria. However, microbial inactivation efficiency depends on processing parameters (power, frequency, temperature, and time) as well as intrinsic product properties such as pH and composition. Low pH conditions generally improve microbial sensitivity, whereas higher solid content can reduce the inactivation efficiency. Under sublethal temperature conditions (<50 °C), ultrasound alone generally results in limited inactivation, typically below 1 to 2 logs even with extended treatment times. Microbial resistance is influenced by structural characteristics, with yeasts and molds often exhibiting greater resistance under mild processing conditions [6], [57], [58].

The effectiveness of ultrasound processing is influenced by the characteristics of the treated system. Components such as suspended solids and dissolved macromolecules can modulate cavitation behavior and mass transfer, thus affecting overall inactivation performance [59]. Although this aspect was not explicitly addressed in the present study, it should be considered in optimizing processing conditions.

From an engineering perspective, ultrasound also offers advantages in processes such as emulsification, where cavitation-induced shear forces enable the formation of fine and stable dispersions with improved interfacial properties. Despite these benefits, industrial implementation remains limited due to challenges related to scalability and sonicator design. Recent advances in ultrasonic equipment, including improved horn configurations, have enhanced the feasibility of scaling up high-intensity ultrasound processes, indicating increasing potential for industrial applications [60], [61].

## 3 Methodology and numerical simulation

A cylindrical flow-through sonicator was selected due to its potential to generate a uniform acoustic field concentrated along the central flow-through zone, enabling efficient energy transfer and compatibility with continuous processing conditions [22], [62]. The energy transfer efficiency in a cylindrical sonicator depends on the coherent interaction between the structural vibration modes of the vessel and the acoustic modes of the enclosed fluid. Among these interactions, flexural modes play a dominant role, as effective coupling is achieved when the transverse mechanical impedance of the structure matches the acoustic impedance of the fluid. Such impedance matching facilitates constructive interference and enhances acoustic energy propagation within the cavitation zone. The dynamic behavior of the structure is governed by the loading of the fluid, which alters the natural frequencies of the lower-order modes. The structural design methodology has been presented in previous work [63]. The breathing mode condition, in which the longitudinal wavelength of the cylinder is equal to the circumference and the bending wavelength exceeds the acoustic wavelength of the fluid, provides the criteria for strong constructive interference between axial and radial fluid oscillations. The realization of this coupling depends on the careful selection of wave numbers, geometric dimensions, and material parameters that govern the resonant interaction between the structure and the fluid.

**Fig. 1:**
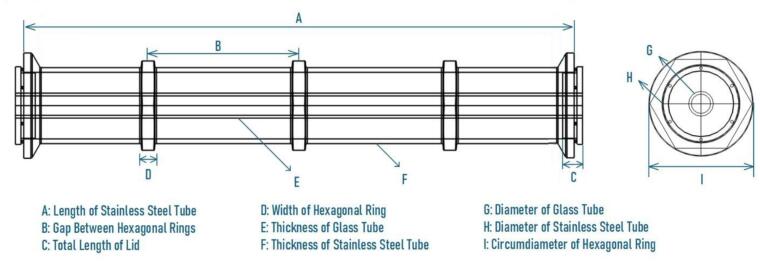
Design parameters in juice pasteurization sonicator.

These key design parameters are illustrated in Fig. 1, which presents the geometric and structural features responsible for improving acoustic energy transfer. The system consists of a cylindrical stainless-steel structure that incorporates three hexagonal rings positioned at defined intervals, an inner glass tube, and two end plates. Eighteen sonotrodes are mounted symmetrically on hexagonal rings to ensure a uniform acoustic energy distribution within the sonicator volume. The stainless-steel housing was developed through structural–acoustic optimization, accounting for multiple mechanical and acoustic parameters. The configuration is also designed to ensure that the inner glass tube, incorporated for hygienic operation, experiences a highly concentrated sound pressure field along the central axis. The dimensions of the inner glass tube and the integration of the actuators significantly influence the acoustic field distribution and, consequently, the efficiency and structural resilience of the sonicator.

The vibrational and acoustic behavior of the coupled system is validated using three-dimensional finite element modeling in COMSOL Multiphysics. This simulation confirms the theoretical design assumptions and supports the optimization of the sonicator configuration. The effectiveness of the structural modeling approach has previously been validated [63]. The glass inner tube and end-lids are specifically designed to enable pasteurization experiments while maintaining a strongly focused acoustic field within the sonicator core. Excitation of ideal vibration modes enhances energy transfer from the transducers to the liquid phase.

The acoustic pressure field in the cavitating liquid was described using the frequency-domain Helmholtz equation, obtained from the small-amplitude acoustic wave equation under harmonic excitation. Since the presence of bubbles modifies both sound propagation and acoustic attenuation, the pressure amplitude P was formulated in terms of a complex effective wavenumber $k_{m}$, following the linearized bubbly-liquid formulation of Prosperetti and Commander and Prosperetti [64], [65]:

$$\nabla^{2}P+k_{m}^{2}P=0,$$

where the real part of $k_{m}$ represents the modified sound propagation in the bubbly liquid, while its imaginary part accounts for attenuation caused by bubble oscillations. In the absence of bubbles, $k_{m}$ reduces to the real wavenumber k=ω/c, where ω is the angular frequency and c is the speed of sound in the homogeneous liquid.

The complex wavenumber is given by

$$k_{m}^{2}=\frac{\omega^{2}}{c^{2}}+4\pi \omega^{2}\int_{0}^{\infty }\frac{R_{0}f\left(R_{0}\right)}{\omega_{0}^{2}-\omega^{2}+2ib\omega }\;dR_{0},$$

where $R_{0}$ is the equilibrium bubble radius, $f(R_{0})$ is the bubble-size distribution, $\omega_{0}$ is the natural angular frequency of the bubble, and b is the damping coefficient.

The thermophysical and acoustic properties used in the numerical model are summarized in Table 1. Pure-water and bubbly-water parameters were adopted from acoustic-cavitation modeling data, whereas the fruit juice properties were selected from reported thermophysical data for apple juice and comparable fruit-juice systems [66], [67], [68], [69], [70], [71]). In the absence of direct data for bubbly apple juice, bubbly-water parameters were used as first-order approximations for cavitation-related quantities, including active bubble radius (7–10μm), bubble number density ($1.0\times 10^{10}$ m^-3^), and bubble volume fraction ($1.4\times 10^{-5}$. It should be noted that the speed of sound in a bubbly liquid is frequency-dependent; therefore, the value of $884\;m\;s^{-1}$ corresponds specifically to the excitation frequency of 29.7kHz used in the simulation.

**Table 1:** Thermophysical and acoustic properties of pure water, bubbly water, and apple juice [66], [67], [68], [69], [70], [71].

**Property**	**Pure water**	**Bubbly water**	**Apple juice**
Density, ρ (kg m^−3^)	998	998	1046
Speed of sound, c (m s^−1^)	1481	884	1543
Surface tension, σ (N m^−1^)	0.0725	0.0725	0.064
Dynamic viscosity, μ (Pa s)	$8.90\times 10^{-4}$	$8.90\times 10^{-4}$	0.050
Heat capacity, $c_{p}$ (J kg^−1^ K^−1^)	4182	4182	3860

In the flow-through sonicator, hydrodynamic cavitation can also be utilized. A multi-hole nozzle is installed along the flow path, inducing a localized pressure drop at each orifice. This pressure reduction promotes bubble nucleation and enhances cavitation efficiency. The design principles and performance characteristics of such orifice nozzles have been investigated in previous studies [72]. At the applied mass flow rate (36  L/min), the five-hole Venturi-shaped nozzle generates a flow velocity in the narrow section approximately 5.6  m/s. This results in a cavitation number that is not sufficiently low to independently induce fully developed transient cavitation. However, in combination with a pulsating acoustic field (> 300 kPa), cavitation activity is significantly enhanced, promoting the initiation and collapse of larger bubbles, as well as increased turbulence and mixing of the apple juice. The positioning of the Venturi relative to the cavitation field is optimized and validated through simulation.

## 4 Materials and methods

Fresh Ingrid Marie apples were purchased from a local supplier and processed for juice extraction. The extracted juice was packaged using an industrial hydraulic fruit juice press and subsequently frozen as soon as possible to minimize microbial growth and preserve the integrity of the sample prior to analysis.

The samples were collected every 15 min over a total processing time of 1 h (with additional sampling at 5 and 10 min in the final treatment), immediately placed on ice and subsequently frozen. The cavitation zone in the flow circuit of the sonicator system accounts for 17 % of the total volume, implying that effective processing is limited to this fraction (0–600 s). The sonicator was cleaned between tests with 0.05 % NaOH in Milli-Q water and rinsed twice with pure water.

For yeast and mold growth analysis, Petrifilm plates (Neogen, UK) were used. The samples were incubated for 5 days at 23 ${\;}^{{}^{\circ}}$ C and evaluated in terms of colony growth.

To ensure accuracy and external validation of the microbial analysis, the untreated sample (blank) and some of the most effective treated samples, selected based on the Petrifilm results, were sent for detailed microbial analysis (yeast, mold, and aerobic microorganisms at 30 ${\;}^{{}^{\circ}}$ C) to Eurofins Food & Feed Testing Sweden (Jönköping).

In addition, scanning electron microscopy (SEM) was performed on two imaging scales (200 and 500  μ m) to qualitatively assess the evolution of the surface morphology as a function of the ultrasound treatment time. Also, sedimentation behavior was monitored 10 days after treatment.

The performance of the system was assessed on the basis of yeast and mold inactivation as the primary pasteurization indicator. The microbial growth before and after treatment was quantified using Petrifilm plates and the results were expressed as colony reduction after incubation. Data were further analyzed with respect to treatment time, temperature range, and excitation mode to evaluate the effectiveness of sonication conditions.

## 5 Experimental procedure

**Fig. 2:**
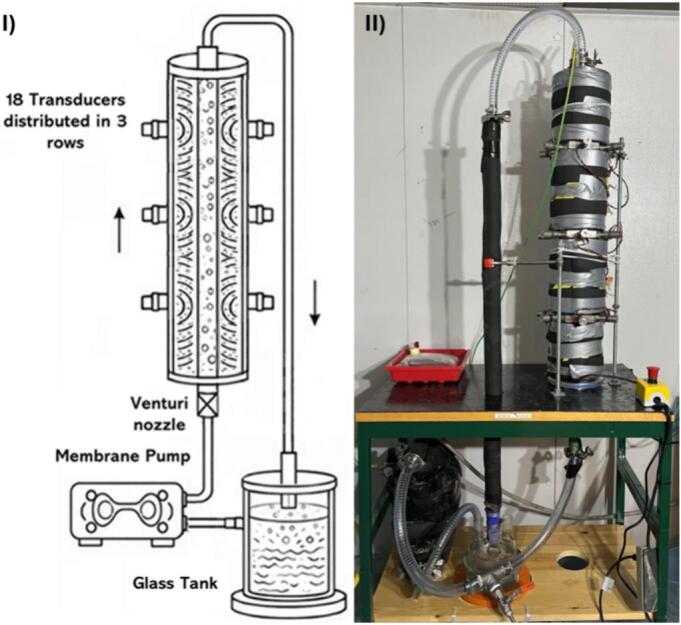
Fruit juice pasteurization sonicator: (I) Schematic diagram; (II) Fabricated device.

The graphical schematics of the sonication systems designed and fabricated for pasteurization are presented in Fig. 2. The design parameters for fruit juice pasteurization are defined in Table2.

**Table 2:** Geometrical parameters and finalized values used in the fruit juice sonicator.

**Symbol**	**Description**	**Value (mm)**
A	Length of Stainless Steel Tube	998
B	Gap Between Hexagonal Rings	273.5
C	Total Length of Lid	36
D	Width of Hexagonal Ring	25
E	Thickness of Glass Tube	5
F	Thickness of Stainless Steel Tube	8.5
G	Outer Diameter of Glass Tube	45
H	Outer Diameter of Stainless Steel Tube	137
I	Circumdiameter of Hexagonal Ring	186

The transducers were operated at approximately 27.7 kHz (Chirp, frequency bandwidth 800 Hz, square wave) as the primary frequency and 42.3 kHz (pure square wave) as the secondary frequency, with an acoustic input power of 300–350 W. Experiments were conducted within temperature ranges of 30–40 °C, 40–45 °C, 45–50 °C, and 50–55 °C in both single and dual-frequency excitation modes. The applied treatments and operating conditions are summarized in Table3.

**Table 3:** Experimental ultrasound conditions used for fruit juice treatment.

Treatment	Frequency (kHz)		Power (W)	Temperature (°C)
2–3	$f_{1}$	$f_{2}$		
SF-1	(28.1–27.3)	−	300	30–40
DF-1	(28.1–27.3)	42.3	300	30–40
SF-2	(28.1–27.3)	−	300	40–45
DF-2	(28.1–27.3)	42.3	300	40–45
DF-3	(28.0–27.2)	42.2	300	45–50
DF-4	27.6	43.7	350	50–55

For the higher-temperature conditions (DF-3 and DF-4), external heating was applied to maintain the desired temperature range. Therefore, in these cases, the temperature increase was not solely attributed to sonication, unlike in the preceding treatments. Since all treatments were performed using preheated liquid, they are generally considered thermosonication processes.

Pure thermal pasteurization controls were not performed at the same temperature ranges used in the experiments. However, all treatment temperatures were below 60 °C, a range in which thermal pasteurization alone is generally not expected to produce substantial microbial inactivation under comparable processing times. Therefore, the observed microbial reduction is considered to result primarily from the combined action of cavitation and mild heating, rather than from temperature alone.

In the dual-frequency configuration, the excitation frequencies were alternated between adjacent transducers so that every other transducer in each row operated at a different frequency. As a result, nine transducers operated at the lower frequency and nine at the higher frequency.

The selected flow velocity was determined based on prior experimental and numerical investigations of hydrodynamic and acoustic cavitation for process intensification in cellulose fibrillation, particularly in flow-through sonicators incorporating Venturi-type nozzles [45].

In all tests, a 5-hole Venturi nozzle was implemented to combine hydrodynamic and acoustic cavitation effects. The hydrodynamic power, based on a RMS pressure difference of 72  kPa was approximately 47  W. Therefore, a compromise was adopted between maximizing cavitation intensity and maintaining stable hydrodynamic conditions.

The selected flow rate ensured a reproducible cavitation regime while avoiding instabilities and large air bubbles that could alter local pressure distributions and cavitation behavior.

Flow conditions were qualitatively validated by audible acoustic emissions associated with cavitation, which correlate with cavitation intensity [73], [74].

To isolate the effects of thermal conditions and intensity of acoustic cavitation, the flow parameters were kept constant throughout the experiments.

This approach is consistent with previous studies that emphasize the importance of decoupling multi-parameter interactions, particularly temperature, acoustic power, and flow-induced turbulence, for systematic optimization of energy efficiency [75], [72].

**Fig. 3:**
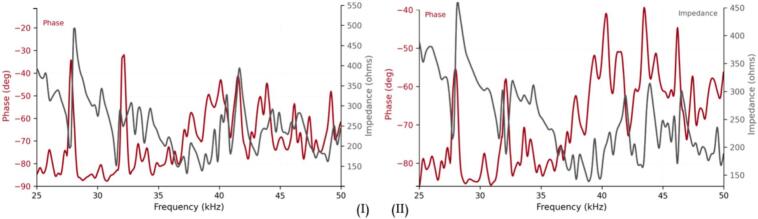
Electrical impedance and phase measurements of different transducer configurations: (I) nine transducers operating at 27 kHz and (II) nine transducers operating at 42 kHz.

The adequacy of the operating conditions was further supported by electrical impedance measurements of the sonication system. A reduction in impedance at excitation frequencies indicated an improved coupling between transducers and fluid, associated with enhanced acoustic energy transfer and cavitation activity[76]. The electrical impedance and phase spectra for different transducer configurations are shown in Fig. 3, where nine transducers operate at 27 kHz and the remaining nine at 42 kHz.

## 6 Results and discussion

### 6.1 Structural–acoustic simulation

The simulation results confirm that the structural–acoustic design objectives were achieved, with a strong concentration of sound pressure within the inner glass tube and relatively uniform axial wave propagation. This behavior reflects the effective coupling between the structural vibration modes and the acoustic modes of the fluid.

Several potential resonance frequencies were identified in the numerical model of the sonicator. Among them, the selected excitation frequency of 29.7  kHz produced the highest average sound pressure level in the flow-through zone, together with uniform vibrational mode shapes in the cylindrical structure, hexagonal rings, and actuators. The predicted mode shape and pressure distribution demonstrate efficient energy transfer and spatial focus of the acoustic field. Constructive interference patterns are observed along the sonicator length, whereas a high-intensity region is maintained within the inner glass tube, supporting effective cavitation conditions.

The displacement field indicates a well-defined structural vibration pattern consistent with the excitation of the targeted modes, ensuring stable acoustic coupling and sustained pressure amplification.

In addition to acoustic effects, the five-hole Venturi nozzle introduces hydrodynamic contributions to cavitation by inducing localized flow acceleration and pressure drop, and with a hydraulic diameter of approximately 1.5  mm and a Reynolds number of $4.3\times 10^{3}$, the flow is in the transitional regime, promoting turbulence and pressure fluctuations. However, the onset of cavitation depends primarily on the local pressure relative to the vapor pressure. Under these conditions, the hydrodynamic pressure drop is close to, but not always sufficient for, sustained cavitation. The acoustic field further reduces local pressure during the rarefaction phases, lowering the cavitation threshold. As a result, the combined hydrodynamic and acoustic effects enhance the initiation of cavitation and the activity of bubbles near the orifice [72].

**Fig. 4:**
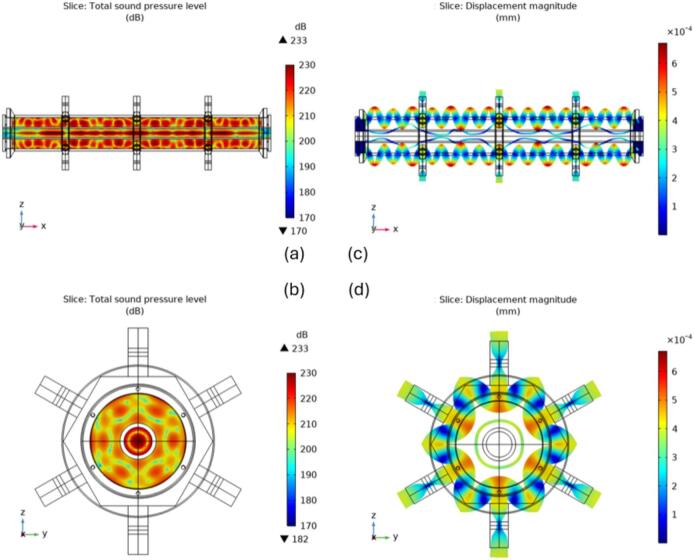
The pressure distribution (a & b) and vibrational mode shapes (c & d) at 29.7 kHz and power of 300 W.

**Fig. 5:**
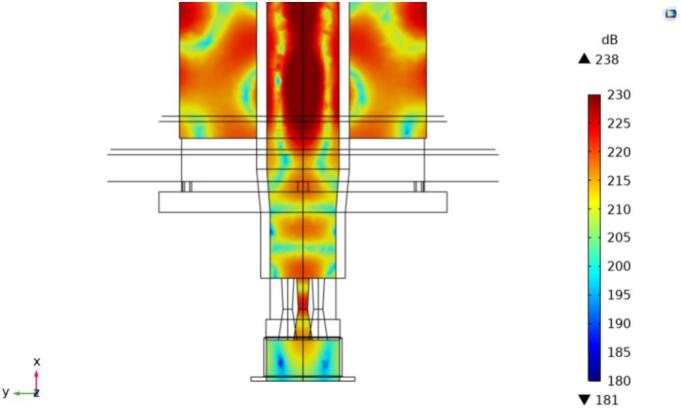
The pressure fluctuation (Sound Pressure Level [dB]) inside the Venturi-shaped nozzle at the vicinity of resonance frequency of sonicator.

Fig. 4 shows the pressure distribution in the target resonance mode, while Fig. 5 presents the corresponding pressure fluctuations within the orifice region.

### 6.2 Acoustic pressure spectral analysis

**Fig. 6:**
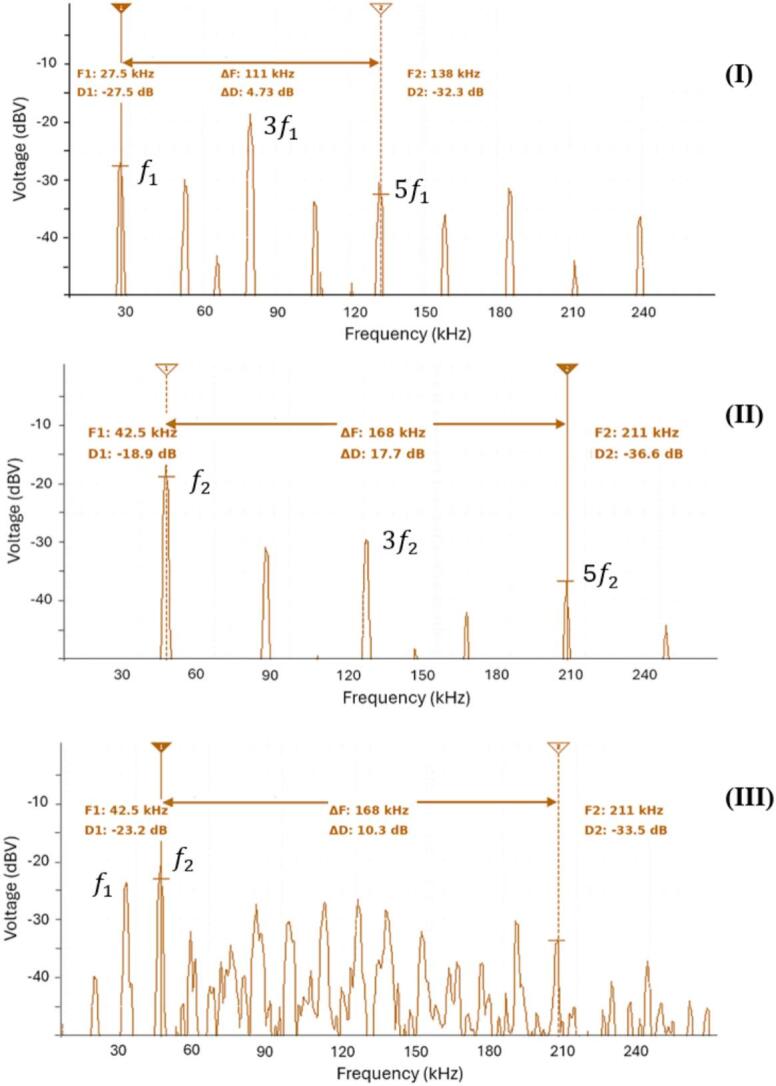
Pressure spectra: (I) nine transducers under single-frequency excitation at 27 kHz; (II) nine transducers under single-frequency excitation at 42 kHz; (III) eighteen transducers under dual-frequency excitation.

The acoustic pressure spectra were measured under single- and dual-frequency excitation conditions. Fig. 6 presents the frequency-domain response for two configurations of nine transducers operating at approximately 27  kHz (I) and 42  kHz (II), as well as the combined dual-frequency excitation of 18 transducers (III). The pressure transducer was positioned within the glass tube in the center of the sonicator. The water was kept in steady-state conditions (no flow) and the temperature was controlled at 45 ${\;}^{{}^{\circ}}$ C.

In single-frequency cases (150  W), distinct peaks are observed at the fundamental frequencies ($f_{1}$ and $f_{2}$), accompanied by pronounced odd harmonics (e.g. $3f_{1}$, $5f_{1}$ and $3f_{2}$, $5f_{2}$). At 42  kHz, the pressure response of odd harmonics corresponds to the square wave excitation. However, due to nonlinear effects caused by cavitation and sonicator response, even harmonics and fractional harmonics can also be observed. At 27  kHz, the nonlinear response becomes more pronounced, particularly in the third harmonic, which exceeds the fundamental amplitude by 8.5  dB.

Under dual-frequency excitation (III, 300  W), the pressure spectrum becomes more complex, exhibiting a broader distribution of peaks. In addition to the primary components ($f_{1}$ and $f_{2}$), multiple interaction frequencies and harmonic contributions that overlap are observed. The increased spectral density and the presence of interacting frequency components indicate a higher level of acoustic field complexity, which is associated with enhanced cavitation activity and a more efficient energy distribution within the sonicator. In ultrasonic systems, acoustic pressure amplitudes on the order of 100  kPa or higher are generally associated with the onset of inertial (transient) cavitation; however, this threshold is not fixed and depends strongly on the properties of the liquid, the content of dissolved gas and the excitation conditions. The measured pressure levels in the present system, particularly under dual-frequency excitation, therefore suggest favorable conditions for intensified cavitation dynamics. The FFT spectrum describes the pressure in terms of dBV. The conversion to Pascal was performed using

$$p=SV_{ref}10^{dBV/20},$$

where p is the acoustic pressure in Pa, S is the hydrophone sensitivity (3.56 MPa/V), $V_{ref}=1$ V is the reference voltage, and dBV is the magnitude obtained from the FFT spectrum. The acoustic pressure values for the single- and dual-frequency operations, including their harmonic components, were obtained from the FFT analysis of the measured signals. For single-frequency operation at 27kHz, the acoustic pressures were $p(f_{1})=150.1\;kPa$, $p(3f_{1})=399.4\;kPa$, and $p(5f_{1})=112.6\;kPa$. For the single-frequency operation at 42kHz, the corresponding values were $p(f_{2})=503\;kPa$, $p(3f_{2})=113\;kPa$, and $p(5f_{2})=52\;kPa$. In the dual-frequency mode at 27 and 42kHz, the measured pressures were $p(f_{1})=225\;kPa$, $p(f_{2})=283\;kPa$, and $p(5f_{2})=75\;kPa$.

### 6.3 Microbial inactivation

**Fig. 7:**
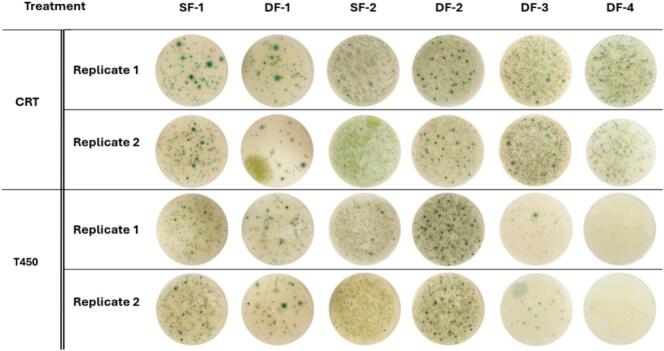
Petrifilm yeast and mold count plates for samples treated for 450  s under different sonication conditions, compared with the untreated control (CRT), after 3 days of incubation. The inoculated circular area had an approximate diameter of 4.5  cm.

An initial evaluation of microbial inactivation was performed using Petrifilm plates to assess yeast and mold growth under different treatment conditions. As illustrated in Fig. 7, the inactivation performance varies between the treatments applied. At lower temperature intervals (30–40c), no reduction trend is observed. At mid temperature interval (40–45c) a consistency increase was observed at the dual-frequency setting. Although DF-3 and DF-4 demonstrate a substantial decrease in colony formation after 450  s.

The growth behavior in DF-2 treatment can be attributed to suboptimal thermo-acoustic conditions. Moderate temperature levels, when not sufficiently elevated, can fall within the microorganism growth range and, in combination with insufficient acoustic intensity, can stimulate microbial activity rather than induce lethal effects [77]. Under such sublethal conditions, ultrasound can enhance mass transfer and nutrient availability, thus supporting microbial survival or even apparent increases in colony counts.

In contrast, the enhanced performance observed in DF-3 and DF-4 can be explained by the achievement of more effective thermo-acoustic conditions. The combination of ultrasound and heat (manothermosonication) has been reported to significantly improve microbial inactivation when both factors are applied at sufficient intensity, while sublethal regimes (manosonication) result in only limited reduction [6].

Consequently, the improved results in DF-3 and DF-4 suggest an increase in acoustic field complexity due to intensified cavitation effects under dual-frequency excitation, leading to a more effective microbial disruption.

**Fig. 8:**
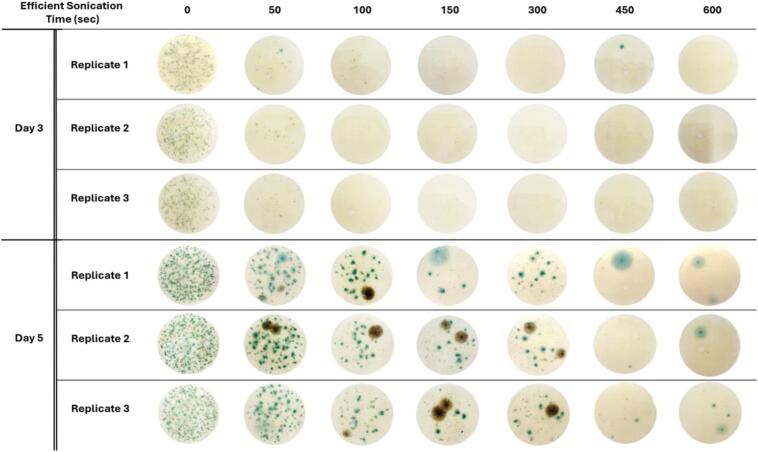
Petrifilm yeast and mold count plates at different efficicent sonication times (0–600 s) after 3 & 5 days in treatment DF-4, showing three replicates. The inoculated circular area had an approximate diameter of 4.5 cm.

The time-dependent behavior of microbial inactivation is further illustrated in Fig. 8 for DF-4 treatment DF-4. A progressive reduction in colony density is observed with increasing sonication time, particularly after 300 s, with near to complete inactivation achieved at 450 and 600 s. The consistency across replicates confirms the stability and reproducibility of the DF-4 condition. In contrast to Fig. 7, which highlights differences between treatment conditions at a fixed time, Fig. 8 demonstrates the temporal evolution of inactivation, emphasizing the critical role of exposure time in achieving effective microbial reduction.

In low-temperature experiments, increased variability in pasteurization performance was observed as a result of temperature-dependent changes in cavitation dynamics and sonicator response. Lower temperatures increase cavitation intensity through increased viscosity and reduced vapor pressure, leading to stronger bubble collapse and improved mechanical effects, resulting in more variable microbial inactivation Gevari et al. [37], Gevari et al. [78].

The limited colony formation observed in some cases of DF-4 is most likely attributable to cross-contamination during handling or plating, as Petrifilm systems are highly sensitive and require strictly aseptic conditions. Therefore, the residual presence of the microbial does not necessarily reflect insufficient treatment performance, but rather experimental limitations. The superior performance of DF-4 suggests that optimized multi-frequency excitation enhances cavitation dynamics, resulting in improved energy localization and microbial damage.

The combined observations from Fig. 7, Fig. 8 indicate that both the selection of temperature and the duration of sonication are critical factors governing microbial inactivation. Although only specific treatments (DF-3 and DF-4) achieve significant reductions, extended exposure time under optimized conditions leads to near-complete inactivation.

The microbial inactivation efficiency was quantified in terms of log reduction based on laboratory measurements. As shown in Table4, DF-4 resulted in substantially higher inactivation compared to DF-3 in all microorganism groups. Yeast populations were reduced from 4000 CFU/mL (CFU: colonies forming units) to below the detection limit (<1 CFU / mL), corresponding to a log reduction of at least 3.6. Mold and aerobic microorganisms were reduced by approximately 2.7 and 2.8 log units, respectively. In contrast, DF-3 exhibited lower reductions of 1.70, 1.75, and 1.5 logs for yeast, mold, and aerobic microorganisms, respectively. The logarithmic reduction values of aerobic microorganisms (30 ${\;}^{{}^{\circ}}$ C) were found to be used as an indicator of the overall efficiency of microbial inactivation. Although these microorganisms do not represent specific pathogenic targets, their reduction provides information on the effectiveness of applied treatment conditions.

**Table 4:** Microbial counts and corresponding log reductions after ultrasound treatments.

Microorganism	Condition	Count (CFU/mL)	Log reduction
Yeast	Blank	4000	–
	DF-3	79	1.7
	DF-4	<1	> 3.6
Mold	Blank	909	–
	DF-3	16	1.8
	DF-4	2	2.7
Aerobic (30 °C)	Blank	3700	–
	DF-3	128	1.5
	DF-4	6	2.8

According to established juice processing guidelines, 5-log reduction of target is for pathogenic microorganisms, whereas yeast and mold are primarily associated with spoilage and shelf-life rather than safety criteria. The results obtained in the present study, particularly for DF-4, demonstrate reductions, reaching at least 3.6 log for yeast and 2.5 log for mold.

The reductions obtained for DF-3 are consistent with previous studies, in which ultrasound treatment under sublethal temperature conditions usually results in limited inactivation (below 2 log). In contrast, the significantly higher reductions observed in DF-4 indicate a higher intensity of cavitation and likely synergistic thermal effects.

The higher susceptibility of yeast compared to mold and aerobic microorganisms may be attributed to differences in cellular structure. Yeast cells are generally larger and more exposed to cavitation-induced shear forces, whereas molds and mixed aerobic populations may exhibit greater structural resistance.

Fruit juice, as a complex medium containing suspended solids and macromolecules, can protect microorganisms from cavitation and thermal effects. This may partially explain why the reductions, although significant, did not reach the 5-log target. Nevertheless, achieving up to 3-log reduction in such a system demonstrates the effectiveness of the applied conditions.

### 6.4 Microstructural evolution and stability

**Fig. 9:**
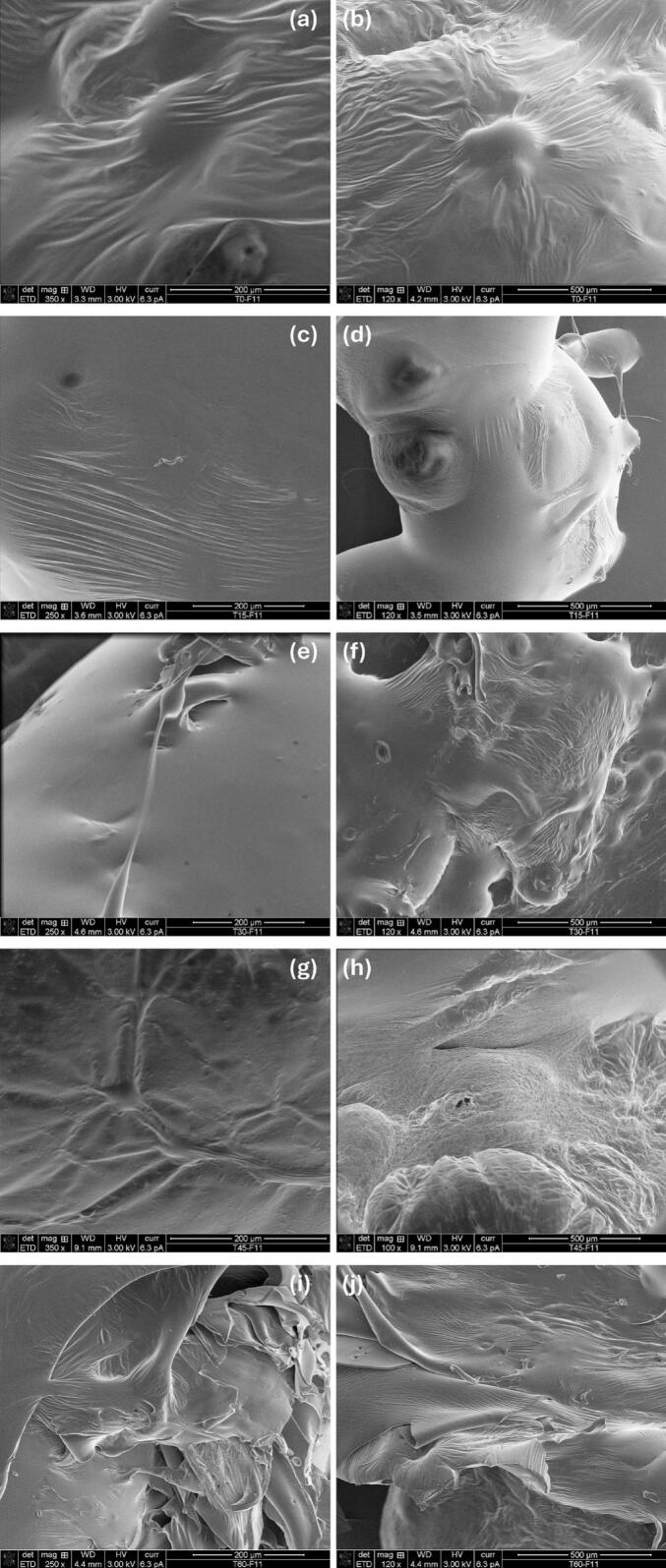
SEM graph of the sample at different ultrasound treatment times (0–600 s) acquired at two imaging scales (200 and 500 μm, respectively). (a,b) t_E = 0 s, (C, D) t_E = 150 s, (E, F) t_E = 300 s, (G, H) t_E = 450 s, and (I, J) t_E = 600 s.

Fig. 9 shows the SEM imaging with different scale bars. Images with a 500μ m scale bar provide an overview of the overall structural changes, while those with a 200μ m scale bar reveal localized surface features and finer morphological details.

The untreated sample ($t_{E}=0\;s$) exhibited a relatively smooth and continuous surface with aligned striations, indicating an intact structural organization. After 150 s of efficient sonication, only minor morphological changes were observed, including slight surface deformation and localized distortion. These early-stage features were relatively subtle and, in some regions, not markedly different from the untreated sample. This behavior can be attributed to the localized and non-uniform nature of cavitation effects at the initial stages of ultrasound exposure, where only specific regions of the surface are affected.

With an increase in efficient treatment time $t_{E}=300\;s$, more pronounced changes became evident, including surface stretching, the formation of irregular features, and localized disruption, indicating a progression of structural modification.

At $t_{E}=450\;s$, the surface exhibited clear wrinkling and the development of a network-like morphology, suggesting intensified mechanical stress and partial structural collapse. The most severe morphological alterations were observed after $t_{E}=600\;s$ efficient time of treatment, where extensive disruption, fragmentation, and loss of surface continuity were evident. These features are consistent with cumulative cavitation effects, including microstreaming, shear forces, and localized pressure fluctuations acting on the material during prolonged exposure.

The analysis is based on qualitative morphological trends rather than quantitative dimensional measurements. Although minor variations in magnification may arise due to differences in the imaging conditions and selected regions, all images were acquired at consistent scales within each set. These variations do not affect the interpretation, as the comparison focuses on general morphological features rather than absolute dimensions.

Representative regions with comparable surface characteristics were selected across all samples, ensuring a consistent basis for comparison. Consequently, the observed trends are considered reliable for evaluating the time-dependent evolution of ultrasound-induced structural modifications.

The SEM results demonstrate a progressive and time-dependent transformation of the surface morphology, with increasing ultrasound exposure leading to enhanced deformation, structural rearrangement, and eventual disruption of the surface architecture.

**Fig. 10:**
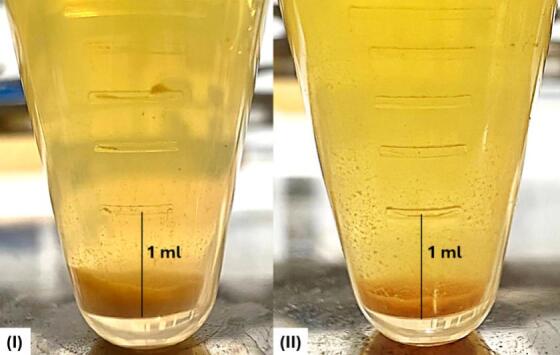
Visual comparison of particle sedimentation behavior in fruit juice samples: (I) untreated sample and (II) after ultrasound treatment (450 s). The marked line indicates the height equivalent to 1 mL of sediment.

As shown in Fig. 10 for a representative case, untreated samples consistently exhibited higher sedimentation compared to ultrasound-treated samples. The untreated sample formed a more compact sediment layer, whereas ultrasound treatment resulted in a less dense sediment and improved particle dispersion within the liquid phase. This behavior can be attributed to ultrasound-induced structural modifications, including reduced particle size and increased surface area, which enhance particle–serum interactions and reduce sedimentation velocity.

Although ultrasound treatment clearly improved suspension stability, no distinct monotonic trend was observed with increasing exposure time. This may be due to the complex interplay between particle fragmentation and aggregation phenomena during sonication, as reported in the literature. In general, the observed reduction in sediment compactness and improved dispersion is consistent with previous studies demonstrating enhanced cloud stability in ultrasound-treated juice systems.

## 7 Conclusion

In this study, a flow-through sonication system combining acoustic and hydrodynamic cavitation was developed and evaluated as a reduced temperature alternative for fruit juice pasteurization. The structural–acoustic design, supported by numerical simulations, enabled effective energy transfer and pressure localization within the sonicator, providing favorable conditions for cavitation activity.

The experimental results demonstrated that microbial inactivation is dependent on the interaction between temperature, excitation mode, and treatment time. Among the investigated conditions, dual-frequency excitation at 50–55 °C (DF-4) showed the highest performance, achieving near-complete inactivation of yeast and substantial reduction of mold and aerobic microorganisms within 450  s with a specific energy consumption of 0.044 kWh/L. As confirmed in this study and according to the literature, the temperature interval 35–40 °C should be avoided in case of sonication with limited power, as the microbial growth can be enhanced.

Acoustic pressure spectral analysis confirmed that dual-frequency excitation generates a broader harmonic content and increased acoustic field complexity, which are associated with enhanced cavitation intensity. These findings were further supported by SEM observations, which revealed a progressive microstructural disruption, including surface deformation and fragmentation with increasing treatment time. In addition, ultrasound treatment improved dispersion stability and reduced sedimentation compared to untreated samples.

Although the achieved microbial reductions did not reach the 5-log target required for pathogenic microorganisms, the results demonstrate that multi-frequency cavitation significantly enhances inactivation efficiency under mild thermal conditions. The observed limitations are attributed to factors such as energy dissipation within the system and matrix-related protective effects.

The results obtained in this study demonstrate that the integration of multi-frequency acoustic excitation with hydrodynamic cavitation can enhance microbial inactivation under reduced-temperature conditions while preserving structural and physical quality attributes. Further improvements in acoustic energy transfer and process optimization are expected to increase inactivation efficiency and support the development of scalable industrial applications.

## CRediT authorship contribution statement

**Sara Maghami:** Writing – original draft, Visualization, Validation, Software, Methodology, Investigation, Formal analysis, Data curation, Conceptualization. **Kimmo Rumpunen:** Writing – review & editing, Visualization, Methodology, Conceptualization. **Örjan Johansson:** Writing – review & editing, Validation, Supervision, Resources, Project administration, Methodology, Investigation, Funding acquisition, Formal analysis, Conceptualization.

## Declaration of competing interest

The authors declare that they have no known competing financial interests or personal relationships that could have appeared to influence the work reported in this paper.
